# Genetic Variants of the Receptor for Advanced Glycation End-products in Susceptibility to Type 2 Diabetes Mellitus in Primary Hypertensive Patients

**DOI:** 10.1038/s41598-017-17068-9

**Published:** 2017-12-08

**Authors:** Hualing Yang, Yangyang Nie, Zhenyi Chen, Linyang Ye, Qingxiang Wang, Zhanxiang Wang

**Affiliations:** 1 Department of Anesthesiology, The First Affiliated Hospital of Xiamen University, Xiamen, Fujian China; 2 The First Clinical Medical College, Fujian Medical University, Fuzhou, Fujian China; 3 Department of Neurosurgery, The First Affiliated Hospital of Xiamen University, Xiamen, Fujian China

## Abstract

Diabetes mellitus is frequently comorbid with hypertension, which is approximately twice as common as diabetes mellitus in China. We designed a case-control association study to inspect the susceptibility of the receptor for advanced glycation end-products (*RAGE*) gene 6 variants to type 2 diabetes mellitus (T2DM) in 2199 patients with primary hypertension (1252 diabetic cases and 947 nondiabetic controls). The genotypes/alleles of −429T > C and 82Gly > Ser variants differed significantly between the two groups, and their associations with T2DM were significant after Bonferroni correction. Two variants, −374T > A and I/D, showed only marginal associations with T2DM. Haplotype analysis of above 4 significant variants indicated that a low-penetrance haplotype simultaneously bearing −429C and 82Ser alleles was overrepresented in cases relative to controls (4.75% vs. 1.72%, *P* < 0.001). Moreover, the predictive capability of 6 variants was significantly superior to available risk factors, with better goodness-of-fit. A predictive nomogram of 4 baseline risk factors and 2 variants of statistical significance was structured, with a good predictive accuracy (C-index = 0.761, *P* < 0.001). Taken together, our findings highlighted a contributory role of the *RAGE* gene, especially its two functional variants −429T > C and 82Gly > Ser, in susceptibility to T2DM in primary hypertensive patients, which may aid early detection and risk assessment for high-risk individuals.

## Introduction

Diabetes mellitus is frequently comorbid with hypertension, and each condition worsens the other^1,2^. In China, nearly a quarter of hypertensive patients concurrently suffer from diabetes mellitus, as projected by a national survey on outpatients^3^ and a large community-based population from Shanghai^4^. China’s national statistics documented that the prevalence of diabetes mellitus and hypertension was 9.7%^5^ and 29.6%^6^, respectively. As we all know, diabetes mellitus and hypertension are major risk factors for cardiovascular diseases, and their comorbidity can engender more serious atherosclerotic events than anticipated on either condition alone^7^. So, early detection and risk assessment of diabetes mellitus in hypertensive patients may represent a more immediate challenge for physicians worldwide.

The ligands-RAGE pathway may serve as a bridge between diabetes mellitus and hypertension in biological mechanisms. The term ‘RAGE’ is the acronym for the receptor for advanced glycation end-products. The RAGE is multi-ligand cell surface receptor, and its ligands include advanced glycation end-products, high mobility group protein box-1, S100 protein family and fibrillar protein aggregates. The RAGE activation by these ligands can activate signal transduction pathway of nuclear factor kappa B (NF-кB) and downstream signaling cascades, leading to a plethora of pro-inflammatory and pro-fibrotic cellular responses through various downstream pathways^8–10^. It is widely recognized that vascular inflammation is one of the common molecular mechanisms in diabetes mellitus and hypertension^11–13^. In addition, an *in-vitro* study demonstrated that the RAGE activation can also account for many features of vascular remodeling diseases, such as vascular smooth muscle cell proliferation and resistance to apoptosis, lending support for a great therapeutic role of RAGE in the treatment of vascular remodeling diseases^14^. So, it is reasonable to anticipate that some genomic alterations in the gene encoding RAGE may be commonly associated with the risk of both diabetes mellitus and hypertension. Current literature is teeming with hundreds of articles inspecting the susceptibility of the *RAGE* gene to diabetes mellitus and its vascular complications^15–17^, with only one study addressing the susceptibility of a promoter locus in hypertensive patients^18^. To gain a deeper insight about the *RAGE*-diabetes mellitus relationship in hypertensive patients, a case-control association study was undertaken and 6 variants in the *RAGE* gene were determined in 1252/947 type 2 diabetic/nondiabetic patients with primary hypertension. Meanwhile, the predictive capability of the *RAGE* genetic variants for type 2 diabetes mellitus (T2DM) risk was evaluated. Finally, a nomogram incorporating baseline risk factors and variants of statistical significance was structured to aid early detection and risk assessment of T2DM for routine clinical practice.

## Results

### Baseline Characteristics

Of 2199 patients with primary hypertension, 1252 cases were diagnosed to have T2DM and 947 controls had normal fasting glucose levels. Cases and controls had analogous distributions in age, sex composition, body mass index (BMI), systolic blood pressure (BP) and diastolic BP, as shown in Table 1. As expected, fasting serum glucose levels were remarkably higher in cases than in controls (140.94 mg/dL vs. 99.18 mg/dL, *P* < 0.001). For blood lipid profiles, mean levels of high-density lipoprotein cholesterol (HDLC) (*P* < 0.001) and apolipoprotein-A (*P* < 0.001) were significantly higher in controls than in cases, whilst mean levels of apolipoprotein-B were significantly higher in cases than in controls (*P* = 0.014). However, mean levels of blood urea nitrogen (BUN) and creatinine were nonsignificant between cases and controls (*P* > 0.2).

**Table 1:** Baseline characteristics of the study population.

Baseline characteristics	Diabetic cases (n = 1252)	Nondiabetic controls (n = 947)	*P*
Age (years)	60.02 (9.79)	59.83 (10.36)	0.499
Sex (Male/Female)	791/461	631/316	0.094
Body mass index (kg/m^2^)	25.25 (3.54)	25.18 (3.32)	0.408
Systolic blood pressure (mm Hg)	135.26 (16.30)	134.86 (11.61)	0.542
Diastolic blood pressure (mm Hg)	86.52 (14.79)	86.98 (9.06)	0.338
Triglyceride (mg/dL)	226.68 (136.36)	225.79 (146.10)	0.867
Total cholesterol (mg/dL)	148.49 (68.06)	145.79 (63.03)	0.353
High-density lipoprotein cholesterol (mg/dL)	56.84 (30.94)	64.19 (33.64)	<0.001
Low-density lipoprotein cholesterol (mg/dL)	93.97 (39.83)	91.65 (34.03)	0.203
Fasting serum glucose (mg/dL)	140.94 (73.62)	99.18 (66.24)	<0.001
Apolipoprotein-A (mmol/L)	1.14 (0.27)	1.23 (0.35)	<0.001
Apolipoprotein-B (mmol/L)	0.78 (0.38)	0.73 (0.40)	0.014
Blood urea nitrogen (mmol/L)	6.0 (4.7, 9.7)	5.9 (4.8, 8.0)	0.882
Creatinine (μmol/L)	89 (73, 137)	87 (71, 176)	0.214

Data are expressed as mean (standard deviation) or median (interquartile range) or count.

### Association of RAGE Gene with T2DM

Six variants in the *RAGE* gene were in the Hardy-Weinberg equilibrium at a significance level of 5%. Both counts and frequencies of three genotypes and mutant allele of six variants are summarized in Table 2. After the conservative Bonferroni correction, genotype distributions differed significantly between cases and controls for −429T > C (*P* < 0.001), I/D (*P* = 0.006) and 82Gly > Ser (*P* = 0.001) variants. In contrast, the frequencies of mutant alleles of −429T > C and 82Gly > Ser variants were remarkably higher in cases than in controls (*P* = 0.009 and <0.001, respectively). The genotypes and alleles of −374T > A variant showed only marginal significance in distributions between cases and controls (*P* = 0.027 and 0.020, respectively). The genotypes and alleles of 1704G > T and 2184 A > G variants did not differ significantly between the two groups.

**Table 2:** Differences in the genotypes and alleles of the *RAGE* gene six variants and their risk prediction for type 2 diabetes mellitus in primary hypertensive patients.

Variants	Region	Genotype or allele	Cases (n = 1252)		Controls (n = 947)		*P*	Genetic model	OR; 95% CI; *P*
			Count	Percent	Count	Percent			
−429T > C (rs1800625)	Promoter	TT	688	54.95%	533	56.28%	<0.001	Additive model	1.35; 1.03–1.76; 0.029
		TC	398	31.79%	343	36.22%		Dominant model	1.21; 0.83–1.78; 0.318
		CC	166	13.26%	71	7.50%		Recessive model	1.76; 1.28–2.41; 0.001
		C (minor)	730	29.15%	485	25.61%	0.009		
−374T > A (rs1800624)	Promoter	TT	690	55.11%	551	58.18%	0.027	Additive model	1.35; 1.03–1.79; 0.032
		TA	441	35.22%	334	35.27%		Dominant model	1.29; 0.88–1.89; 0.184
		AA	121	9.66%	62	6.55%		Recessive model	1.44; 1.02–2.04; 0.040
		A (minor)	683	27.28%	458	24.18%	0.020		
I/D (−407 to −345)	Promoter	II	1001	79.95%	772	81.52%	0.006	Additive model	1.24; 1.01–1.51; 0.037
		ID	208	16.61%	163	17.23%		Dominant model	1.14; 0.90–1.44; 0.281
		DD	43	3.43%	12	1.21%		Recessive model	3.04; 1.64–7.05; 0.001
		D (minor)	294	11.74%	187	9.87%	0.049		
82Gly > Ser (rs2070600)	Exon-3	GG	524	41.85%	459	48.47%	0.001	Additive model	1.29; 1.12–1.48; <0.001
		GA	553	44.17%	395	41.71%		Dominant model	1.36; 1.12–1.64; 0.002
		AA	175	13.98%	93	9.82%		Recessive model	1.49; 1.11–2.00; 0.008
		A (minor)	903	36.06%	581	30.68%	<0.001		
1704G > T (rs184003)	Intron-7	GG	638	50.96%	504	53.22%	0.562	Additive model	1.19; 0.86–1.65; 0.288
		GT	535	42.73%	384	40.55%		Dominant model	1.18; 0.98–1.43; 0.082
		TT	79	6.31%	59	6.23%		Recessive model	0.89; 0.61–1.31; 0.562
		T (minor)	693	27.68%	502	26.50%	0.387		
2184 A > G	Intron-8	AA	821	65.58%	620	65.47%	0.115	Additive model	1.05; 0.90–1.23; 0.536
		AG	347	27.72%	282	29.78%		Dominant model	0.96; 0.79–1.17; 0.693
		GG	84	6.71%	45	4.75%		Recessive model	1.63; 1.08–2.46; 0.020
		G (minor)	515	20.57%	372	19.64%	0.448		

Abbreviations: OR, odds ratio; 95% CI, 95% confidence interval. Adjustment was performed for the additive, dominant and recessive models by considering age, sex, body mass index, high-density lipoprotein cholesterol, apolipoprotein-A and apolipoprotein-B.

After adjusting for age, sex, BMI, HDLC, apolipoprotein-A and apolipoprotein-B, the prediction of the *RAGE* gene 6 variants for T2DM risk was explored under the additive, dominant and recessive models, respectively (Table 2). In detail, −429T > C variant was strongly associated with T2DM under the recessive model (odds ratio (OR): 1.76; 95% confidence interval (95% CI): 1.28–2.41; *P* = 0.001) model. The −374T > A’s association was only marginally significant under the additive and recessive models. For I/D variant, the recessive model exhibited the strongest risk magnitude (OR: 3.40; 95% CI: 1.64–7.05; *P* = 0.001). The 82Gly > Ser variant was consistently associated with the significant risk of T2DM under all three genetic models (OR: 1.29, 1.36 and 1.49; 95% CI: 1.12–1.48, 1.12–1.64 and 1.11–2.00; *P* < 0.001, 0.002 and 0.008, respectively for the additive, dominant and recessive models). None or marginal significance was seen for 1704G > T and 2184 A > G variants under all three models.

### Haplotype Analysis

As the *RAGE* gene four variants (−429T > C, −374T > A, I/D and 82Gly > Ser variants) were in significant association with T2DM, a haplotype analysis was done based on the four variants. The frequencies (>1%) of estimated haplotypes were compared between cases and controls (Table 3). The most common haplotype was T-T-I-G (alleles in order of −429T > C, −374T > A, I/D and 82Gly > Ser variants), and its frequency was significantly higher in controls than in cases (46.17% vs. 36.59%, *P* < 0.001). Another haplotype C-T-D-A was contrastingly overrepresented in cases than in controls (4.75% vs. 1.72%, *P* < 0.001). When the most common haplotype was regarded as a reference group, haplotype C-T-D-A was significantly associated with 1.99-fold increased risk (95% CI: 1.15–3.44), while the association between haplotype T-T-I-A and T2DM was only marginal (OR: 1.30; 95% CI: 1.02–1.67). There was no detectable significance for the other haplotypes in Table 3.

**Table 3:** Common haplotype frequencies of the *RAGE* gene four significant variants between diabetic cases and nondiabetic controls in primary hypertensive patients and their risk prediction estimates.

Haplotypes	Diabetic cases	Nondiabetic controls	*P*	Simulated *P*	OR (95% CI)
T-T-I-G	36.59%	46.17%	<0.001	<0.001	Reference group
T-T-I-A	22.92%	20.50%	0.073	0.075	1.30 (1.02–1.65)
C-A-I-G	14.99%	13.04%	1.000	1.000	1.27 (0.92, 1.67)
C-A-I-A	8.18%	8.27%	0.147	0.150	1.30 (0.99–1.71)
T-T-D-G	5.57%	5.42%	0.651	0.651	1.20 (0.87–1.63)
C-T-D-A	4.75%	1.72%	<0.001	<0.001	1.99 (1.15–3.44)
C-A-D-G	2.33%	2.28%	0.274	0.268	1.26 (0.79–2.00)
C-T-I-G	1.55%	1.56%	0.246	0.248	1.28 (0.69–2.38)

Abbreviations: OR, odds ratio; 95% CI, 95% confidence interval. In a haplotype, four alleles were arranged in order of −429T > C, −374T > A, I/D and 82Gly > Ser variants. Only haplotypes with estimated frequencies over 1% in both cases and controls were listed and compared between the two groups.

When considering all possible haplotypes as a whole, its association with available baseline risk factors was provided in both cases and controls (Table 4). Only HDLC was significantly associated with the omnibus haplotype in both cases (Global statistic: 35.33; *P* = 0.009) and controls (Global statistic: 27.82; *P* = 0.011). The association for the other baseline risk factors remained nonsignificant.

**Table 4:** The association of baseline characteristics with derived haplotypes based on the *RAGE* gene four significant variants* in both diabetic cases and nondiabetic controls in primary hypertensive patients.

Characteristics	Diabetic cases		Nondiabetic controls	
	Global statistic	*P*	Global statistic	*P*
Age (years)	18.81	0.172	6.58	0.764
Sex	16.83	0.207	−2.58	1.000
Body mass index (kg/m^2^)	5.27	0.982	6.36	0.848
Systolic blood pressure (mm Hg)	17.73	0.220	15.55	0.158
Diastolic blood pressure (mm Hg)	9.84	0.774	10.54	0.483
Triglyceride (mg/dL)	1.96	1.000	10.45	0.402
Total cholesterol (mg/dL)	16.70	0.213	16.45	0.125
High-density lipoprotein cholesterol (mg/dL)	35.33	0.009	27.82	0.011
Low-density lipoprotein cholesterol (mg/dL)	−103.02	1.000	−97.95	1.000
Apolipoprotein-A (mmol/L)	15.51	0.277	10.98	0.445
Apolipoprotein-B (mmol/L)	15.49	0.278	9.81	0.548
Blood urea nitrogen (mmol/L)	13.73	0.393	2.35	0.997
Creatinine (μmol/L)	6.93	0.906	5.12	0.925

*The four significant variants included −429T > C, −374T > A, I/D and 82Gly > Ser in the *RAGE* gene as shown in Table 3.

### Predictive Capability

**Figure 1:**
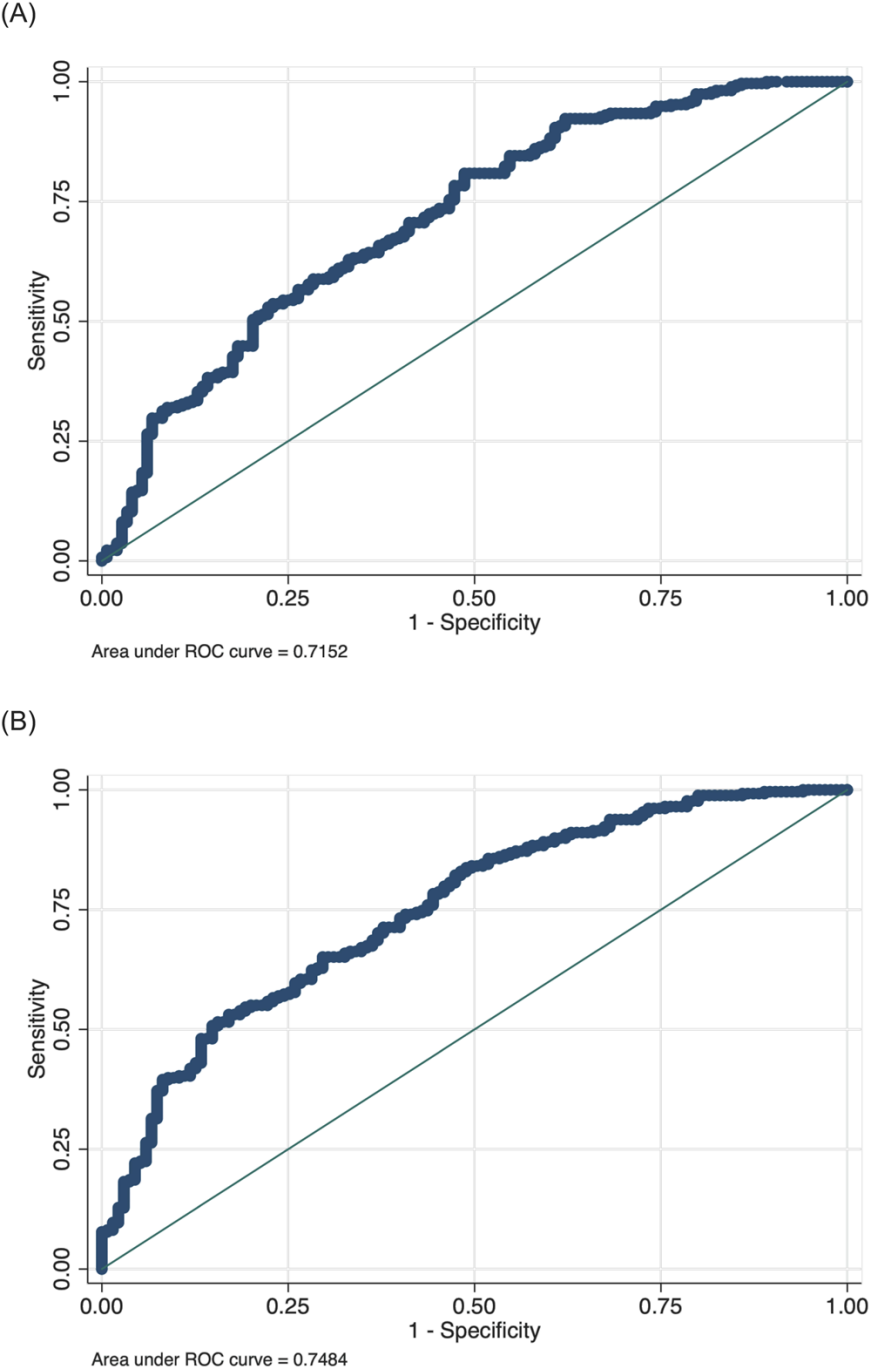
The receiver-operating-characteristic (ROC) curves based on available risk factors (**A**) and additional six variants in the *RAGE* gene (**B**).

Predictive capability of the *RAGE* gene 6 variants for T2DM in primary hypertensive patients was assessed by the receiver-operating-characteristic (ROC) analysis (Fig. 1). Regressing available baseline risk factors yielded an area under the ROC curve of 0.715 (95% CI: 0.666 to 0.773) (Fig. 1A), which was increased to 0.748 (95% CI: 0.698 to 0.799) after adding the *RAGE* gene 6 variants to baseline regression model (Fig. 1B), the difference being marginally significant (*P* = 0.044). Both baseline model and modified model had better goodness-of-fit, as reflected by the Hosmer-Lemeshow test (*P* = 0.183 and 0.352, respectively).

### Predictive Nomogram

**Figure 2:**
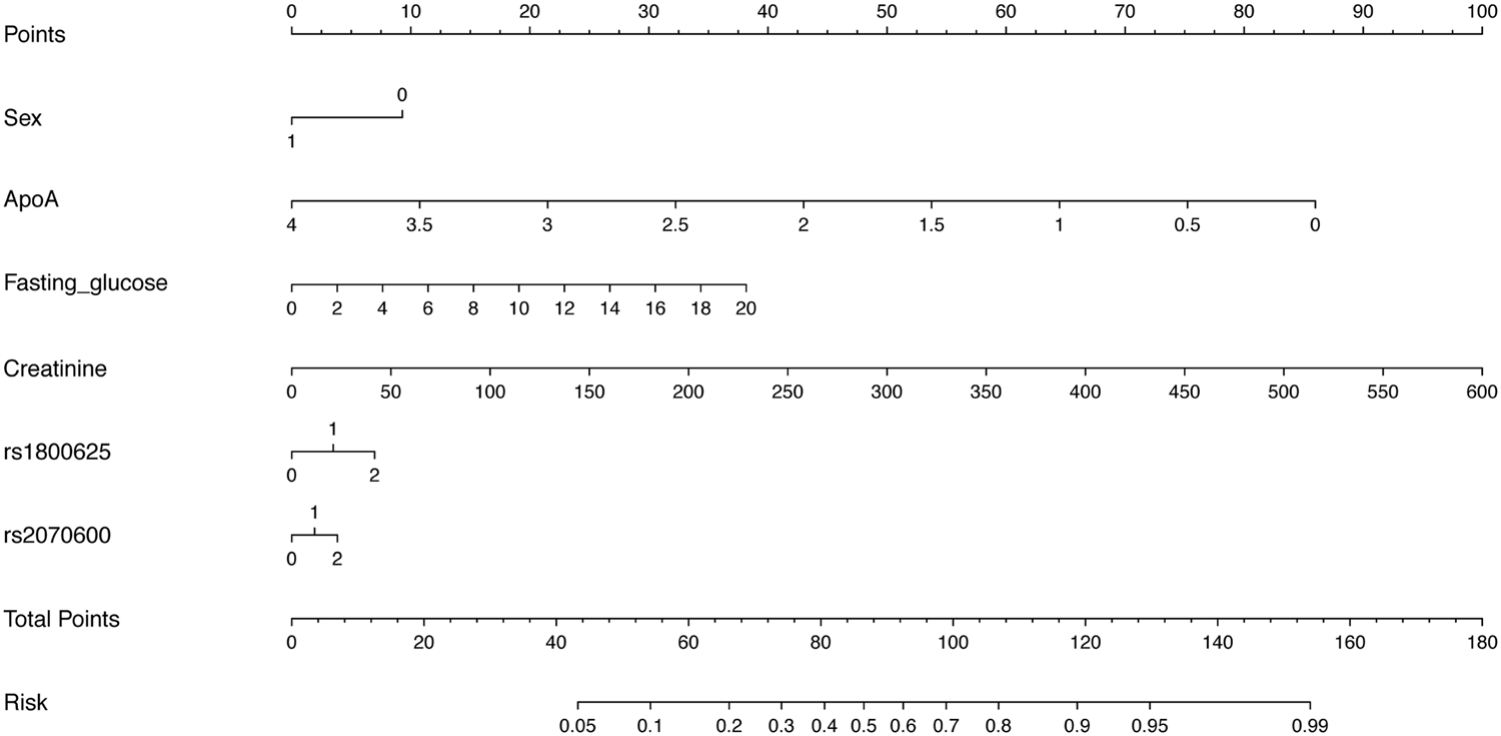
Predictive nomogram of all significant risk factors and genetic variants in the *RAGE* gene for the risk of having type 2 diabetes mellitus in primary hypertensive patients. Abbreviations: ApoA, apolipoprotein-A; Fasting_glucose, fasting serum glucose. Risk magnitude can be calculated by projecting a vertical line from the value of each attribute to the “Points” row at the top of this nomogram, and record the score. The scores of all attributes are summed to produce a total point score and mark this score on the “Total Points” row, and the projected point on the “Risk” row at the bottom is predicted risk estimate.

To determine which risk factors or *RAGE* genetic variants can exert a major role in risk prediction, a Forward regression analysis was undertaken. Four risk factors (sex, apolipoprotein-A, fasting serum glucose and creatinine) and two variants (−429T > C and 82Gly > Ser) were associated with the significant risk of T2DM in primary hypertensive patients. A predictive nomogram based on these six attributes is exhibited in Fig. 2. The predictive accuracy of this nomogram was good and the C-index, equivalent to the area under the ROC curve, was 0.761 (*P* < 0.001), suggesting 76.1% correct model identification of the high-risk patients with primary hypertension comorbid with T2DM across all possible pairs of patients.

## Discussion

The prevalence of diabetes mellitus is escalating annually worldwide^5,19^. When diabetic patients have comorbid hypertension, more intensive treatment is required to prevent vascular-associated morbidity and mortality. In China, hypertension is approximately twice as common as diabetes mellitus, and one in four hypertensive patients has diabetes mellitus^3–5,20^. It might therefore be more practical to identify and separate high-risk individuals who could benefit from timely medical intervention to prevent diabetes mellitus developing in hypertensive patients. As both diabetes mellitus and hypertension are complex polygenic disorders^21,22^, it will be a breakthrough for the characterization of common genetic determinants associated with both conditions. Hence, we chose the *RAGE* gene as a research target in view of its prominent regulatory role in vascular inflammation, a possible shared mechanism between diabetes mellitus and hypertension.

In this present study, we genotyped the *RAGE* gene six variants in 2199 patients with primary hypertension to refine risk assessment for T2DM via a case-control study design. On the one hand, we found that the mutation of two variants, one in the promoter region (−429T > C) and one in exon 3 (82Gly > Ser), can significantly increase the risk of experiencing T2DM in primary hypertensive patients. In support of this finding, −429T > C and 82Gly > Ser variants were found to be significantly associated with the development of vascular-associated complications in patients with diabetes mellitus^23,24^. By contrast, two meta-analyses failed to observe any significance for −429T > C and 82Gly > Ser variants, as well as −374T > A and 1704G > T variants with the risk of diabetes mellitus^15,25^, which might be explained in part by the differences in risk factor exposure, ethnogeography and genetic background across races and ethnicities. There have been numerous candidate gene studies to show that some genetic variants may be risk factors for diabetes mellitus in one ethnic group, but remained controversial in other ethnic groups^26,27^. It is worth noting that no study had took the comorbidity of hypertension into account when inspecting the association between the *RAGE* gene and diabetes mellitus risk. Growing evidence has disclosed the potentials of soluble forms of RAGE as novel therapeutic targets of arterial stiffness and hypertension^28–30^. Lack of consideration for hypertension thus might lead to a selective misclassification of high-risk individuals that can cloud the potential association between the *RAGE* gene and diabetes mellitus susceptibility. Our findings for the first time reported that the *RAGE* gene two functional variants, −429T > C and 82Gly > Ser, were independently associated with the significant risk of T2DM in primary hypertensive patients. Importantly, both variants were observed to add significantly to the predictive capability of T2DM over available baseline risk factors in this study. For clinical applicability of our findings, the mutation of −429T > C and 82Gly > Ser variants in the *RAGE* gene, along with other four risk factors as illustrated in the predictive nomogram (Fig. 2), can aid improving risk assessment with individualized close monitoring of hypertensive patients at risk for T2DM.

On the other hand, we inspected the combined role of the *RAGE* gene four significant variants in susceptibility to T2DM by a haplotype analysis. Our findings indicated that with the except of the commonest haplotype, only a low-penetrance haplotype simultaneously bearing −429C and 82Ser alleles was overrepresented in diabetic cases relative to nondiabetic controls, suggesting a possible interaction of the two variants in the sense of synergy. Our further omnibus haplotype-phenotype analysis revealed that the derived haplotypes as a whole, exerted a significant impact on plasma HDLC, especially in patients with comorbid T2DM and hypertension. Although haplotype analysis is thought to be more powerful than individual variant analyses^31^, it is not without its limitations because it often requires a very large study population and its results are difficult to explain. As diabetes mellitus is multifactorial in nature, it is likely that multiple variants of candidate gene synergize in determining the disease risk^32^. Future studies involving dense coverage of the *RAGE* genomic variants and large same sizes are clearly warranted to unveil the genetic underpinnings of the *RAGE* in the development of T2DM in primary hypertensive patients.

Finally, there are several limitations for this study. The first limitation was that an observed association between the *RAGE* gene and T2DM did not prove cause, and so additional prospective and experimental studies will be needed to provide proof of causation. Moreover, considering sample sizes involved, our findings needed confirmation in adequately powered studies. The second limitation was that this study was carried out in a selected group of primary hypertensive patients referred to a university affiliated hospital, and it was unclear whether our findings would be applicable to other populations. The third limitation was that data on physical activity and lifestyle factors were not collected, which might result in unaccounted residual confounding. The fourth limitation was that most of our primary hypertensive patients aged over 50 years, and it was of interest to inspect the association of the *RAGE* gene with T2DM in a younger population because genetic factors may play a dominant role in young-onset T2DM. The fifth limitation was the limited coverage of the *RAGE* genomic variants, and it was unclear whether other variants in linkage disequilibrium with the two significant variants detected in this study determined T2DM risk. Also, 6 variants selected for analysis were based on previous genetic association studies, and we acknowledged that other variants not tested might provide additional information. The sixth limitation was that soluble forms of RAGE were not available for us to analyse their relationship with the *RAGE* gene in susceptibility to T2DM. Finally, our study population consisted of only Han Chinese, hence its generalizability may be limited.

Despite above limitations, the present findings collectively highlighted the contributory role of the *RAGE* gene, especially its two functional variants −429T > C and 82Gly > Ser, in susceptibility to T2DM in primary hypertensive patients. Our findings are of much clinical significance, as we provide baseline evidence that primary hypertensive patients positive for −429C and 82Ser alleles presumably need closer monitoring for early detection and risk assessment of diabetes mellitus in the daily clinical routine.

## Methods

The ethics committee of the First Affiliated Hospital of Xiamen University reviewed and approved the study protocol. In addition, we can confirm that all methods were performed in accordance with the relevant guidelines and regulations.

### Study Patients

The present study was carried out from May 2014 to March 2017 in a total of 2199 patients diagnosed with primary hypertension (1422 males and 777 females) aged 31 to 86 years. All patients were of Han Chinese origin, and they were diagnosed and recruited from the First Affiliated Hospital of Xiamen University, Xiamen city, Fujian province, China. Of them, 1252 patients were comorbid with T2DM as the ‘diabetic cases’ and 947 patients were confirmed to have no clinical indication of T2DM as the ‘nondiabetic controls’, who had no personal or family history of diabetes mellitus among first-degree relatives. All patients read and signed informed consent files for the collection and subsequent use of their blood samples.

### Clinical Diagnosis

BP was measured twice by trained physicians or nurses when subjects had rested for at least 5 minutes. Primary hypertension is diagnosed with an average systolic BP reading of >140 mmHg or an average diastolic BP reading of >90 mmHg or the use of antihypertensive medications, and importantly secondary causes of hypertension are excluded after an extensive biochemical and clinical examination. Meanwhile, fasting serum glucose was assayed in all primary hypertensive patients, and some of them had received oral glucose tolerance test (OGTT). T2DM is diagnosed if fasting serum glucose ≥7.0 mmol/L or 2-hour OGTT glucose ≥11.1 mmol/L.

### Laboratory Assay

Before clinical examination, all primary hypertensive patients were invited to answer a structured questionnaire about their medical histories and lifestyles. Meanwhile, body weight and height were measured on site and taken to calculate BMI. Afterwards, a 5-mL venous blood sample was drawn from all patients for further biomedical examination and genetic analysis.

Besides fasting serum glucose, other biomedical markers including triglyceride, total cholesterol, HDLC, low-density lipoprotein cholesterol (LDLC), apolipoprotein-A, apolipoprotein-B, BUN and creatinine were assayed subsequently at the Laboratory Medicine Centre of The First Affiliated Hospital of Xiamen University.

### Genotyping Assay

Six genomic variants were selected from the *RAGE* gene based on current literature, including −429T > C (promoter), −374T > A (promoter), the −407^th^ to −345^th^ fragment insertion/deletion (I/D) (promoter), 82Gly > Ser (exon 3), 1704G > T (intron 7) and 2184 A > G (intron 8) variants^15,23,33,34^. Genomic DNA was prepared from 200 μl of peripheral blood lymphocytes for all samples by aid of the QIAamp DNA Mini kits (Qiagen) at the university laboratory. Based on a case-control study design, genomic DNA samples of 1252 diabetic cases and 947 nondiabetic controls were subjected to the TaqMan genotyping assay (Applied Biosystems, Inc, Foster City, CA, USA). Experimental protocols are available upon request.

### Statistical Analyses

All baseline data were typed into a computerized database and were independently cross-checked for any data entry mistakes. Comparisons of continuous data between two groups were done by the Student’s t-tests, and that of quantitative data were done by the Person χ^2^ contingency tables. Each of the *RAGE* gene 6 variants was tested to see whether the observed genotype frequencies were concordant with the expected Hardy-Weinberg proportions by the Person χ^2^ or the Fisher’s exact contingency tables. Genotype and allele frequencies of each variant were calculated, and their differences between cases and controls were determined by the Person χ^2^ contingency tables. Adjusted ORs with 95% CIs were derived from the multivariate Logistic regression analysis to quantify the association magnitude between the *RAGE* gene and T2DM risk in primary hypertensive patients. Moreover, the frequencies of haplotypes based on 4 significant variants were estimated by using the program Haplo.Stats, version 1.7.7 developed by Sinnwell and Schaid (http://www.mayo.edu/research/labs/statistical-genetics-genetic-epidemiology/software). The omnibus association of derived haplotypes as a whole with each baseline characteristic was also examined by the program Haplo.Stats.

The ROC curves were used to assess the relative predictive capability of *RAGE* genomic variants beyond that of available baseline risk factors. Areas under the ROC curves were compared accordingly. Meanwhile, the Hosmer-Lemeshow test was used to test the goodness-of-fit for risk prediction models.

Finally, a predictive nomogram was structured based on the significant attributes from the Forward Logistic regression analysis of available baseline risk factors and 6 genomic variants in the *RAGE* gene. The nomogram can create a simple graphical representation of a statistical predictive model that generates a single numerical estimate of the probability of a clinical event^35,36^. A nomogram’s predictive accuracy is measured through a concordance index (c-index), which can quantify the level of concordance between predicted probabilities and the actual chance of having the event of interest. Specifically, the c-index denotes the proportion of pairs, with the responders having a higher predicted probability of response than the non-responders^36^.

Statistical analyses were performed with the use of Stata software, version 14.1 (http://www.stata.com) and R language, version 3.3.3 (https://www.r-project.org). All reported *P* values were based on 2-sided tests. Unless otherwise stated, *P* value of less than 0.05 was reported to be statistically significant.

### Availability of data and materials

The datasets used and/or analyzed during the current study are available from the corresponding author on reasonable request.
